# Supplementary material for: Intestinal Barrier Impairment Induced by Gut Microbiome and Its Metabolites in School-Age Children with Zinc Deficiency
Source: Nutrients. 2024 Apr 26;16(9):1289. doi: 10.3390/nu16091289 (PMC11085614; doi:10.3390/nu16091289)
Supplement: Supplementary file 1 [file nutrients-16-01289-s001.zip › Supplemental table.pdf]

Supplemental Table S1. Functional abundance of the Top20 KEGG level 2 pathways.

KEGG level 2 pathways	ZD		CK		*P* value
	Mean	SEM	Mean	SEM	
Protein Families: Signaling and Cellular Processes	60118.4	1126.8	63541.4	630.71	0.926
Protein Families: Genetic Information Processing	55082.9	1281.88	59362.5	781.49	0.194
Carbohydrate Metabolism	35877.8	1770.62	37165.2	585.84	0.994
Protein Families: Metabolism	34291.4	1087.63	35688.1	371.44	0.975
Membrane Transport	26610.9	818.86	31204.3	513.24	0.152
Signal Transduction	27037.7	1043.89	24330.1	666.13	<0.001
Amino Acid Metabolism	22568.3	823.12	24600.5	417.44	0.005
Metabolism of Cofactors and Vitamins	19414.7	890.85	20238.4	477.15	0.944
Cellular Community-Prokaryotes	18170	435.65	19802.7	278.52	0.236
Translation	17197.5	322.47	20370.8	442.96	0.01
Glycan Biosynthesis and Metabolism	16638	992.31	14971.3	379.44	0.003
Energy Metabolism	13721.6	464.95	14077.1	322	0.594
Nucleotide Metabolism	13322.9	394.05	14330.7	291.86	0.163
Replication and Repair	12854.9	173.57	13616.2	228.58	0.916
Lipid Metabolism	9992.4	428.13	10331.8	250.2	0.933
Folding, Sorting and Degradation	9394.5	268.34	10478.3	197.2	0.002
Drug Resistance: Antimicrobial	8882.3	293.2	8683.4	118.45	<0.001
Metabolism of Other Amino Acids	7405.2	281.42	8050.6	164.68	0.13
Biosynthesis of Other Secondary Metabolites	7137.5	299.77	7139.9	134.8	0.209
Cell Growth and Death	6457.9	165.47	5902.4	126.61	<0.001

$P < 0.01$, $P < 0.001$.

Supplemental Table S2. Significant KEGG level 3 pathways between ZD and CK groups.

**KEGG level 3 pathways**	**baseMean**	**log2FoldChange**	***p* value**
Plant hormone signal transduction	29.23899774	1.822873961	<0.001
Steroid hormone biosynthesis	17.56269741	1.791882517	<0.001
Glycosphingolipid biosynthesis - lacto and neolacto series	78.47230789	1.689129402	<0.001
Biosynthesis of 12-, 14- and 16-membered macrolides	26.23829191	1.653803657	0.014
Rheumatoid arthritis	38.66486937	1.567792246	<0.001
Osteoclast differentiation	40.50911777	1.467922599	<0.001
Flavone and flavonol biosynthesis	102.0765839	1.457255311	<0.001
Biosynthesis of enediyne antibiotics	36.90415727	1.377576772	<0.001
Circadian entrainment	15.89953932	1.31170611	0.038
Type I polyketide structures	16.89554529	1.27917982	<0.001
MAPK signaling pathway - fly	26.05939945	1.275089355	0.002
p53 signaling pathway	29.71078869	1.231237704	0.020
Hepatitis C	24.08896864	1.22898501	0.017
Meiosis - yeast	608.3151717	1.198858201	<0.001
Glycosylphosphatidylinositol (GPI)-anchored protein	186.0515401	1.09433301	<0.001
Glycosaminoglycan degradation	940.3339347	1.088974678	<0.001
Fluorobenzoate degradation	39.11897259	1.060913138	0.001
Penicillin and cephalosporin biosynthesis	175.0103432	0.993619581	<0.001
Lysosome	1845.687462	0.992000934	<0.001
Styrene degradation	119.6231309	0.816285148	0.007
Apoptosis - fly	61.72466118	0.807839575	0.016
Glycosphingolipid biosynthesis - ganglio series	466.877759	0.77241972	0.007
Bacterial chemotaxis	1133.389454	0.707147586	0.004
Transcriptional misregulation in cancer	172.6450696	0.691933337	<0.001
Pertussis	737.9949817	0.661217551	0.013
Mannose type O-glycan biosynthesis	113.1915796	0.654292161	0.025
Prion disease	190.1114318	0.648434087	0.001
Shigellosis	58.93671284	0.647631348	0.034
Sphingolipid metabolism	1969.531555	0.643797868	<0.001
Non-alcoholic fatty liver disease	142.7569434	0.637929197	<0.001
Amyotrophic lateral sclerosis	340.8012742	0.620743865	<0.001
Longevity regulating pathway - multiple species	1122.992913	0.599995303	<0.001
Cardiac muscle contraction	147.6023197	0.597693147	0.009
Other glycan degradation	3065.592744	0.583811529	<0.001
Cationic antimicrobial peptide (CAMP) resistance	2571.964656	0.575930208	<0.001
Thyroid hormone synthesis	97.35016205	0.562407394	<0.001
Lipopolysaccharide biosynthesis protein	1905.845278	0.543174575	0.015
CD molecule	275.0326468	0.533933233	0.016
Lipopolysaccharide biosynthesis	2054.37605	0.530623488	0.007
Huntington disease	292.8385966	0.507152698	0.003
Ubiquinone and other terpenoid-quinone biosynthesis	660.0785514	0.458542014	0.024
Glycosphingolipid biosynthesis - globo and isoglobo series	871.091396	0.450737349	0.040
beta-Lactam resistance	3898.562562	0.427838436	<0.001
Protein digestion and absorption	458.5463589	0.386370355	0.035
Cyanoamino acid metabolism	1806.749256	0.379415505	<0.001
NOD-like receptor signaling pathway	534.0137373	0.366361456	<0.001
Transcription machiner	3134.090678	0.356645056	0.001
Antigen processing and presentation	192.2288766	0.352325838	0.029
Pathways of neurodegeneration - multiple diseases	426.645223	0.351325566	0.041
Small cell lung cancer	135.2825236	0.346640529	0.022
Protein phosphatases and associated protein	545.3810115	0.33175052	0.015
Parathyroid hormone synthesis, secretion and action	197.0760696	0.321093792	0.047
Phenylpropanoid biosynthesis	1305.004153	0.318572419	0.003
Spliceosome	486.6262861	0.302432952	0.014
Biosynthesis of various secondary metabolites - part 2	682.2407815	0.3002109	0.002
Gastric cancer	137.9955304	0.296631635	0.031
Fluid shear stress and atherosclerosis	773.1607016	0.294201922	<0.001
Citrate cycle (TCA cycle)	1692.070835	0.291453566	<0.001
Biofilm formation - Pseudomonas aeruginosa	3161.832157	0.285082276	0.016
Pathogenic Escherichia coli infection	379.7796246	0.279876322	0.046
Th17 cell differentiation	183.1955596	0.273811008	0.041
Fanconi anemia pathway	818.9131385	0.271708118	0.010
Prostate cancer	205.7479817	0.267104594	0.026
Antimicrobial resistance gene	4383.405457	0.255227878	<0.001
Two-component system	22298.01833	0.251807483	<0.001
IL-17 signaling pathway	204.2831792	0.251636835	0.043
Lysine degradation	378.284903	0.247039898	0.030
Colorectal cancer	207.4182537	0.241374899	0.030
Progesterone-mediated oocyte maturation	220.7896584	0.24030304	0.024
Pentose and glucuronate interconversions	2985.559847	0.219320404	0.009
Carbon fixation pathways in prokaryotes	2515.066879	0.20685288	0.016
Salmonella infection	665.6936041	0.196899667	0.035
Thermogenesis	660.2691598	0.196357019	0.014
Nitrogen metabolism	1992.320403	0.19159027	0.001
Two-component syste	12702.50677	0.18326973	0.029
Plant-pathogen interaction	994.5192193	0.178886053	0.016
RNA transport	1091.613131	0.17397997	0.007
Peroxisome	786.910544	0.168398397	0.035
O-Antigen nucleotide sugar biosynthesis	3139.291763	0.149450558	0.013
Arginine and proline metabolism	1711.478481	-0.105651115	0.026
Lysine biosynthesis	2846.505281	-0.107803222	0.015
Nucleotide excision repair	2918.887704	-0.122331186	0.041
Pyruvate metabolism	4416.865039	-0.125241875	0.010
Monobactam biosynthesis	850.0710415	-0.125623214	0.024
One carbon pool by folate	2811.24074	-0.126859104	0.022
Glycine, serine and threonine metabolism	3443.355904	-0.131971345	0.046
Glucagon signaling pathway	942.689963	-0.146757508	0.029
Pantothenate and CoA biosynthesis	2019.9159	-0.151543917	0.026
Ribosome biogenesis in eukaryotes	756.6929349	-0.17190119	0.020
Cysteine and methionine metabolism	5920.515669	-0.180811872	0.006
Protein export	3460.48876	-0.181638492	0.031
Streptomycin biosynthesis	2112.257183	-0.18177678	0.001
Acarbose and validamycin biosynthesis	633.191057	-0.19464878	0.005
Inositol phosphate metabolism	754.3250337	-0.195983695	0.023
Prodigiosin biosynthesis	368.3282771	-0.200519216	0.042
Glycerolipid metabolism	2254.974004	-0.204257899	0.001
Selenocompound metabolism	1831.902037	-0.212210216	0.001
Ribosome	10420.12496	-0.214490154	0.037
Ribosom	10420.12496	-0.214490154	0.037
Insulin resistance	651.9703287	-0.222763565	<0.001
Diabetic cardiomyopathy	762.0135933	-0.230995989	0.019
Peptidoglycan biosynthesis	4268.497189	-0.234773772	<0.001
Type II diabetes mellitus	215.1053608	-0.249804805	0.016
Peptidoglycan biosynthesis and degradation protein	5425.058941	-0.262642416	<0.001
Valine, leucine and isoleucine biosynthesis	1787.665205	-0.292500112	0.007
N-Glycan biosynthesis	699.753389	-0.294013418	0.010
Human papillomavirus infection	297.6315322	-0.296140848	0.022
Chloroalkane and chloroalkene degradation	624.9379236	-0.310867273	0.014
RNA polymerase	917.6796533	-0.335793852	<0.001
Vancomycin resistance	3032.597186	-0.35841131	<0.001
Bacterial invasion of epithelial cells	345.6862325	-0.398400493	0.046
Primary bile acid biosynthesis	369.826344	-0.413433351	0.020
Hypertrophic cardiomyopathy	201.375992	-0.436990799	<0.001
Secondary bile acid biosynthesis	635.5351555	-0.478409139	<0.001
Neomycin, kanamycin and gentamicin biosynthesis	623.2884822	-0.478561334	<0.001
Prolactin signaling pathway	167.4342021	-0.483448318	0.001
Biosynthesis of unsaturated fatty acids	352.4939873	-0.486668013	0.001
Ether lipid metabolism	172.7147769	-0.489517328	0.016
Dilated cardiomyopathy	106.3264077	-0.494524166	<0.001
D-Alanine metabolism	1316.9802	-0.496795529	<0.001
Tight junction	238.224345	-0.499979962	0.008
Aminobenzoate degradation	366.2778083	-0.588541533	<0.001
Ubiquitin syste	154.1186377	-0.675000158	0.003
Renin secretion	37.22628646	-0.677139441	0.010
Naphthalene degradation	168.2474856	-0.704282715	<0.001
Domain-containing proteins not elsewhere classifie	74.68550191	-0.714361169	0.043
Phosphotransferase system (PTS)	2376.197674	-0.749884633	<0.001
Chagas disease	107.9047454	-0.795450298	0.006
Cushing syndrome	69.04396598	-0.886302246	0.003
Acute myeloid leukemia	10.0991646	-0.958506444	0.020
Staurosporine biosynthesis	10.86573219	-1.042377953	0.024
Aflatoxin biosynthesis	25.6778879	-1.1573433	0.005
Proteasome	82.93388852	-1.179093339	0.017
Renal cell carcinoma	40.33022464	-1.444942579	<0.001

$P < 0.01$, $P < 0.001$.
