# Supplementary figures and images for: Intestinal Barrier Impairment Induced by Gut Microbiome and Its Metabolites in School-Age Children with Zinc Deficiency
Source: Nutrients. 2024 Apr 26;16(9):1289. doi: 10.3390/nu16091289 (PMC11085614; doi:10.3390/nu16091289)

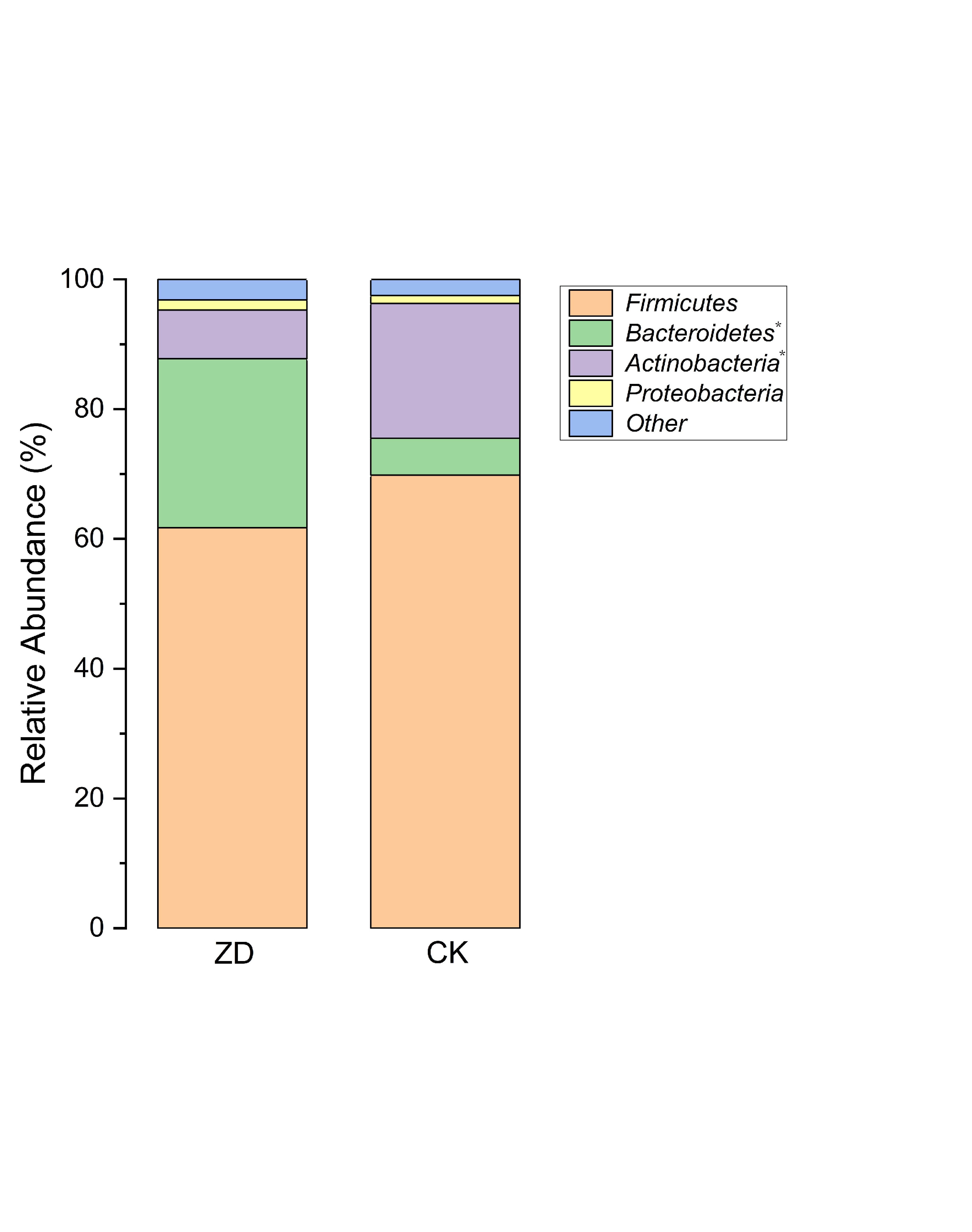

Supplement: Supplementary file 1 [file nutrients-16-01289-s001.zip › Supplemental Figure S1.jpg]

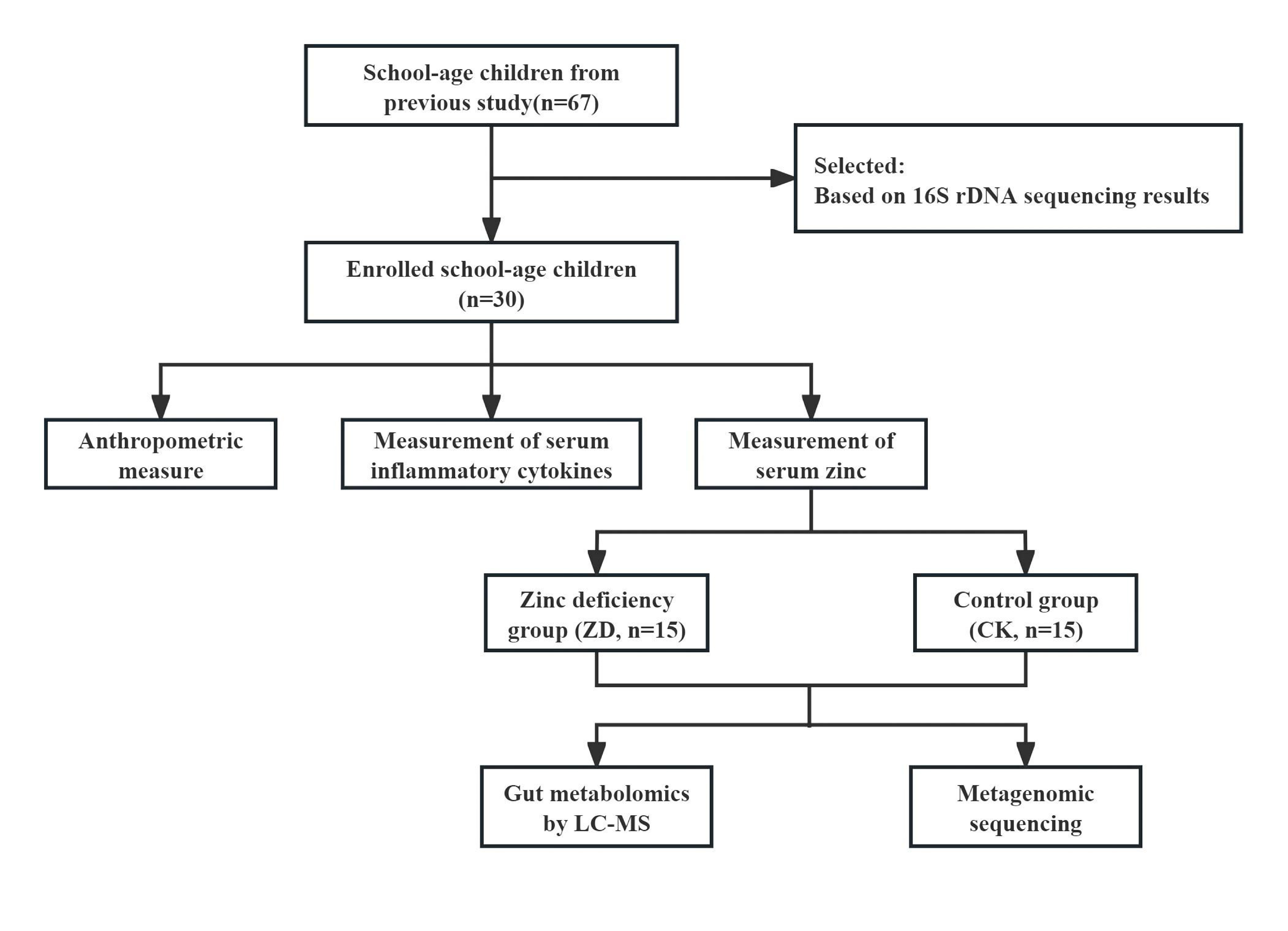

Supplement: Supplementary file 1 [file nutrients-16-01289-s001.zip › Supplemental Figure S4.jpg]
